# Optimization of the Culture Conditions of Lactic Acid Bacteria for Antimicrobial Activity and Mass Production of Cyclic Dipeptides

**DOI:** 10.4014/jmb.2408.08007

**Published:** 2025-06-12

**Authors:** Rui Liu, Gwihwi Shin, Yanggyun No, Jieun Shin, Saouk Kang, Phumbum Park

**Affiliations:** 1 The University of Suwon, Kyonggi-do 445-743, Republic of Korea; 2 Seoul National University, Seoul 08826, Republic of Korea; 3 Irwee Institute, Irwee Co., Ltd., Seongnam 13511, Republic of Korea

**Keywords:** Cyclic dipeptides, *Lactiplantibacillus plantarum*, culture condition optimization, antibacterial activity

## Abstract

Cyclic dipeptides (CDPs) are secondary metabolites from lactic acid bacteria (LAB) known for their antibacterial, antifungal, and antiviral properties. Here, we investigated CDPs to optimize culture media and boost their production in LAB. The optimal conditions and ratio of *Lb. plantarum* LBP-K10 to *Leu. mesenteroides* LBP-K06 (temperature, time, stationary or shaking conditions, carbon or nitrogen source, etc.) were determined through step-by-step optimization. The two strains (K10:K06 = 7:3), cultured together at 30°C for 48 h in stationary conditions, resulted in enhanced antibacterial effectiveness. This was evident against *B. subtilis* ATCC 9372 (clear zone size of 22.6 ± 2.3 mm), *E. coli* S-99 (14.8 ± 1.8 mm), *Salmonella* Gallinarum ATCC 9184 (27.6 ± 2.4 mm), *V. parahaemolyticus* KCTC2471 (13.2 ± 3.2 mm), and *S. aureus* KACC 10768 (12.0 ± 2.1 mm). These results surpassed the clear zone sizes of 13.2 ± 1.4 mm, 12.0 ± 2.2 mm, 19.3 ± 1.0 mm, 9 ± 0.5 mm, and 9.8 ± 0.7 for each corresponding bacterium, respectively, in K10-alone control groups (37°C for 48 h in conventional medium). We further confirmed the production of these cyclodipeptides within cultures. When LBP-K10 and LBP-K06 were co-cultured at a 7:3 ratio under stationary conditions at 30°C for 48 h with 4% sucrose and 1.5% mixed amino acids supplementation, the total production of cyclo(Leu-Pro) and cyclo(Phe-Pro) exceeded that of the LBP-K10 mono-culture by more than 35%, reaching 6.65 ppm compared to 4.91 ppm. These findings hold significant potential for industrial-scale production of CDPs and related products.

## Introduction

Lactic acid bacteria (LAB), especially *Lactiplantibacillus plantarum* and *Leuconostoc mesenteroides*, are gram-positive, facultative, anaerobic, non-spore-forming, heterofermentative bacteria that ferment sugars to produce lactic acid and acetic acid. LAB have consistently demonstrated beneficial effects, such as competitive exclusion of gut pathogens, tumor suppression by cell-mediated immunity, and enhancement of host defenses [1]. Metabolites produced in LAB culture media can inhibit the growth of bacteria and fungi [2, 3]. Among these secondary metabolites produced by LAB, cyclic dipeptides (CDPs) have been intensively investigated recently [4-7].

CDPs are basic cyclic peptides derived from the condensation of two amino acids, featuring a diketopiperazine structure where the piperazine ring's two nitrogen atoms create amide bonds. With the advantages of excellent hydrogen bond formation ability and structural rigidity, CDPs are considered promising alternatives to currently used small bioactive molecules [8-11]. CDPs have been proven to exert important physiological benefits for human health [12], such as reducing alcohol paralysis, fighting memory loss, and inhibiting virally transformed cell growth.

CDPs can be synthesized chemically via amino acid self-condensation or intramolecular cyclization of linear dipeptides [13-15]. However, chemical synthesis requires rigorous conditions to limit racemization and reduce unwanted side reactions. It is well known that various organisms, from mammals to bacteria, can produce CDPs as secondary metabolites or side products of terminal peptide cleavage. Several biological mechanisms of CDP formation pathways have been demonstrated, including non-enzymatic and enzymatic pathways, such as nonribosomal peptide synthetase-mediated or cyclic dipeptide synthetase-mediated synthesis [16-18]. Although several protease enzymes, such as dipeptidyl peptidases [19], have been identified for natural CDP formation, strategies for improving CDP production are still challenged by the complicated bioprocess of CDP formation.

We recently proved that CDPs produced by LAB have antibacterial, antifungal, and antiviral effects. These strains include *Bacillus subtilis* ATCC 9372, *Escherichia coli* S-99 (O157:H7), *S*. Gallinarum ATCC 9184, *Staphylococcus aureus* KACC 10768, and *Vibrio parahaemolyticus* KCTC2471 [20-23]. Increasing the CDP production of the bacteria is required for industrial applications. Thus, the culture conditions for CDP production by *Lb. plantarum* LBP-K10 require further optimization. Apart from *Lb. plantarum* LBP-K10, *Leu. mesenteroides* LBP-K06 is another major microorganism found in Korean kimchi [21]. Although strains LBP-K10 and LBP-K06 belong to two different classifications, they share similar properties, such as acid resistance and colony formation in MRS medium [24]. Moreover, LBP-K10 and LBP-K06 effectively produce quorum-sensing CDP molecules, including cyclo (Leu-Phe), which have cell proliferation activities and antibiotic effects [25, 26]. A co-culture of LBP-K10 and LBP-K06 might significantly improve the growth and production of CDPs compared to LBP-K10 alone.

The co-culture of microorganisms is an effective strategy for activating silent biosynthetic pathways and enhancing the production of cryptic secondary metabolites. Microbial co-culture introduces interspecies interactions, such as competition, synergy, or chemical signaling, which can stimulate the production of novel compounds not observed in mono-cultures. For example, the co-culture of the marine-derived fungus *Penicillium* sp. DT-F29 with *Bacillus* sp. B31 significantly increased the production of prenylated 2,5-diketopiperazines (2,5-DKPs) [27]. This co-culture strategy activated silent gene clusters in *Penicillium* sp., resulting in the isolation of 23 compounds, including 10 novel DKPs. The production of DKPs increased from two in mono-culture to 16 on average in the co-culture. This study highlights how live bacterial interactions are essential for inducing such metabolites, as dead bacteria or their extracts had minimal effects. A broader review of marine microbial co-cultures (2009–2019) reveals diverse structural classes of secondary metabolites, including alkaloids, polyketides, and cyclopeptides [28]. Among these, alkaloids and DKPs were prominent due to their varied biological activities, such as cytotoxicity and antimicrobial properties. Studies on *Penicillium* and *Aspergillus* species have shown that co-culturing can lead to a significant increase in metabolite diversity and yield, underscoring the potential of co-culture strategies for novel drug discovery.

We used a mixed culture of *Lb. plantarum* LBP-K10 and *Leu. mesenteroides* LBP-K06 in the present study and further refined the *Lb. plantarum* LBP-K10 culture conditions to improve CDP production. Carbon and nitrogen source supplementation and temperature optimization were also investigated. As we have shown, within the 17 structures revealed by HPLC analysis of these cyclodipeptide-rich components, cyclo(Leu-Pro) and cyclo(Phe-Pro) formed the two highest peaks on the chromatogram, and both had relatively high concentrations [21, 22]. Therefore, we selected these two substances as our reference compounds to more accurately and efficiently assess the biological activity of the LAB culture.

## Materials and Methods

### LAB Culture Condition Optimization

*Lb. plantarum* LBP-K10 and *Leu. mesenteroides* LBP-K06 were each inoculated into 10 ml of basal medium mMRS (modified MRS;) and cultured at 30°C for 72 h. Different ratios of LBP-K10 and LBP-K06 were mixed and inoculated into 50 ml MRS medium at 1% (v/v) inoculum. Each mixture was cultured in the same medium. The mixed culture and the single cultures were incubated for 72 h at 30°C. After incubation, the supernatants were separated by centrifugation (4,585 × *g*, 20 min; Hanil, Republic of Korea) and refrigerated, and their antibacterial activity was compared. After obtaining the supernatants through centrifugation, we subjected the samples to high-temperature heating (120°C, 2 h) and concentration to eliminate any residual microbial activity [20-23]. Additionally, prior to performing the antimicrobial assays, the samples were filtered to ensure complete removal of any remaining bacterial cells, and the antibacterial activity of the supernatants was compared.

To optimize the incubation time, LBP-K10 and LBP-K06 were co-cultured by inoculating 1% (v/v) in 50 ml of the mMRS medium. The culture temperature was rechecked to optimize the incubation temperature. For conventional LAB, the culture was cultured at 30°C, and the CDP production was confirmed. LBP-K10 and LBP-K06 were each inoculated into 10 ml of the basal medium mMRS and incubated at 30°C for 72 h. Next, the strains were mixed and stationary-cultured by inoculating 1% (v/v) in 50 ml mMRS medium at 30°C for 72 h. After incubation, the supernatant was separated by centrifugation (4,585 × *g*, 20 min) and refrigerated, and the antibacterial activity of the supernatant was compared.

To compare the CDP production results of the stationary and shaking cultures, we inoculated *Lb. plantarum* LBP-K10 and *Leu. mesenteroides* LBP-K06 into 10 ml of mMRS basal medium for the liquid culture. The stationary culture sample was cultured at 30°C in the incubator, and the shaking culture sample was cultured at 30°C in the shaking incubator (LabTech, Republic of Korea) at 150 rpm. Then, LBP-K10 and LBP-K06 were mixed and cultured in 50 ml mMRS medium at 1% inoculum. The mixed culture was divided into stationary and shaking cultures, which were then cultured for 72 h each. After incubation, the supernatants were separated by centrifugation (4,585 × *g*, 20 min) and refrigerated, and their antibacterial activity was compared.

To provide the best conditions for microbial growth and optimize the nitrogen, five nitrogen sources were selected, and the optimal conditions for antibacterial activity were investigated. The medium was prepared by adjusting the concentrations of the yeast extract (Daejung, Republic of Korea), mixed amino acids, soy peptone, tripeptide, and corn steep powder (Kisanbio, Republic of Korea) in 0.5% (w/v) units. LBP-K10 and LBP-K06 were then inoculated into the mMRS basal medium and cultured at 30°C for 36 h. Next, the strains were inoculated at 1% (v/v) in 100 ml of the mMRS medium and cultured at 30°C for 48 h. After incubation, the supernatants were separated by centrifugation (4,585 × *g*, 20 min) and refrigerated, and their antibacterial activity was compared.

To optimize the carbon source, we tested glucose (Duksan, Republic of Korea), D-fructose (Samchun, Republic of Korea), sucrose (Duchefa Biochemical, Republic of Korea), and molasses (Daemyung Chemical, Republic of Korea) based on the formulation with the nitrogen sources mentioned above. The concentrations of glucose and sugar were 1, 2, 3, and 4%, and those of molasses were 2 and 4%. LBP-K10 and LBP-K06 were inoculated separately into the mMRS basal medium and statically incubated at 30°C for 36 h. Next, LBP-K10 and LBP-K06 were statically incubated by mixing 1% inoculum in 100 ml of the mMRS medium at 30°C for 48 h. After incubation, the supernatants were separated by centrifugation (4,585 × *g*, 20 min) and refrigerated, and their antibacterial activity was compared.

### CDP Extraction

The culture broth of the cultured strains was dispensed into sterile 15 ml conical tubes (5 ml each) and centrifuged at 3,500 ×g for 20 min. The supernatants were transferred into sterile 15 ml conical tubes and stored in a refrigerator until further use. A 5-fold ethyl acetate solution (Daejung) was added to the supernatants, and the extraction was carried out at 200 rpm in a shaking incubator at 25°C for 20 h. After extraction, the supernatants of the ethyl acetate mixture were separated using a funnel, and the separated ethyl acetate was evaporated and dissolved in 1 ml of DW.

### High-Performance Liquid Chromatography (HPLC) Analysis

For the analysis of CDPs, including cyclo(Leu-Pro) and cyclo(Phe-Pro), the samples prepared by ethyl acetate extraction were placed in vials at amounts of 200 μl each. The mobile phase consisted of 67% triple distilled water, 3% acetonitrile, and 30% methanol at a flow rate of 0.7 ml/min for 35 min. Before analyzing the samples, the standard was analyzed by concentration, and a standard curve was created. Cyclo(Leu-Pro) and cyclo(Phe-Pro)(Bachem, Switzerland) were used as standard materials.

### Disk Diffusion Assay

*B. subtilis* (ATCC 9372), *E. coli* S-99 (O157:H7), *S*. Gallinarum (ATCC 9184), *S. aureus* (KACC 10768) and *V. parahaemolyticus* (KCTC2471) were used as bacterial pathogen indicators. Each strain was inoculated in 10 ml of liquid medium and cultured at 150 rpm in a shaking incubator at 37°C for 18 h (Table 1), and 1 ml of each culture was suspended in 9 ml of sterile saline. After preparing 100 ml of 1.5% agar medium and cooling it to 50°C, 0.1 ml of each suspended pathogen strain solution was added and mixed by shaking. Then, 15 ml was dispensed into a Petri dish using a disposable pipette and hardened.

Three to four sterile paper discs (8 mm; Whatman, UK) were placed at regular intervals on the agar plate where each indicator pathogen was layered, and 100 μl of the extracted samples were properly added and incubated at 37°C. The size of the clear zones in the culture plates was measured. Average values were calculated from three repeated experiments.

## Results

The LAB culture conditions were optimized to obtain the preferred antibacterial activity, as demonstrated using a disc diffusion assay with cell-free LAB culture broth supernatants. We evaluated several critical factors, including incubation time, temperature, culture method, and nutrient supplementation, using clear-zone size as a measure of antibacterial effectiveness.

### Co-Culture vs. Single Culture

**Fig. 1:**
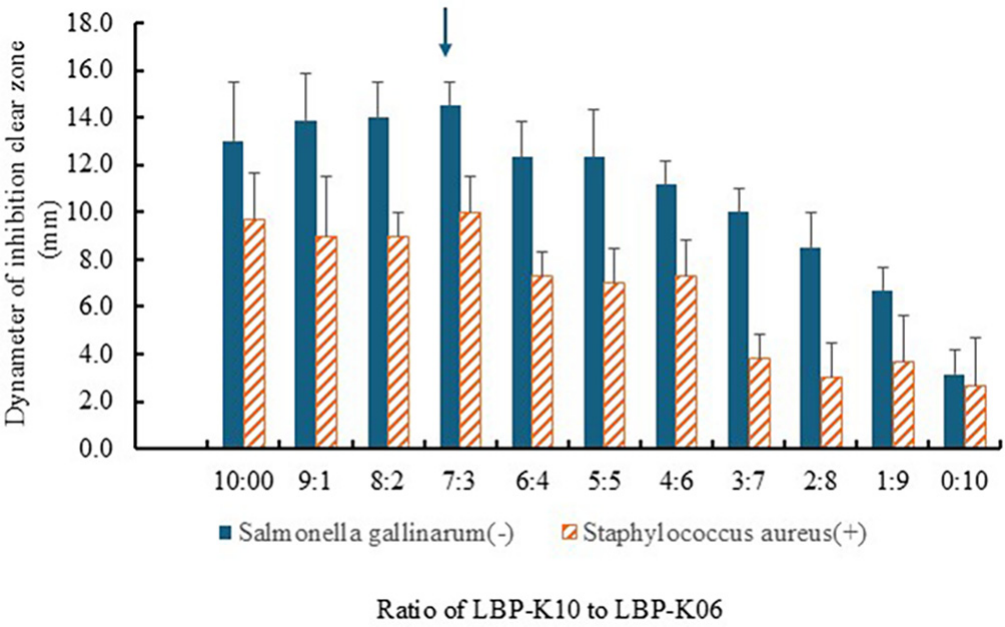
Antibacterial activity depending on the single and the mixed culture samples. Comparative antibacterial activity of conditioned optimized culture broths from *Lb. plantarum* LBP-K10 and *Leu. mesenteroides* LBP-K06 in single and co-culture media. Antibacterial activity was assessed at various time points, revealing no significant differences between the mixed culture conditions overall. However, the largest inhibition clear zone was observed in co-cultures with a 7:3 ratio of *Lb. plantarum* LBP-K10 to *Leu. mesenteroides* LBP-K06 (arrow).

First, we compared the antibacterial activity of the conditioned optimized culture broth obtained from *Lb. plantarum* LBP-K10 and *Leu. mesenteroides* LBP-K06 in the single or series co-culture medium. Though there were no significant differences in antimicrobial activity between the mixed cultures at each time point, and the clear zones of inhibition indicated that the co-cultures of K10 and K06 at the ratio of 7:3 exhibited the largest zone size (Fig. 1).

### Incubation Time

**Fig. 2:**
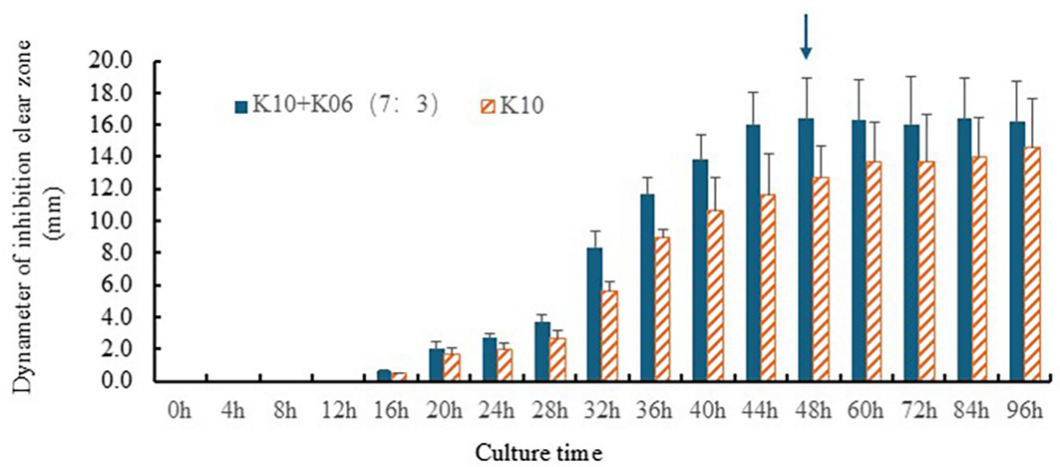
Antibacterial activity depending on culture time. The impact of incubation time (0 to 96 h) on the antibacterial activity of the LAB co-culture broth supernatant (K10 = 7:3) was investigated. Samples were collected at designated time points, and the broth supernatant was analyzed using the disk diffusion method. No inhibitory zones were observed at 0 and 12 h. A distinct inhibitory zone emerged at 24 h, with no significant change in size noted after 48 h (indicated by arrow). Therefore, the optimal incubation time was determined to be 48 h.

Second, the effect of incubation time (0 to 96 h) on the antibacterial activity of the LAB co-culture (K10:K06 = 7:3) broth supernatant was investigated. Mixed culture samples were collected over time, and the culture broth supernatant was subjected to disk diffusion analysis. At 0 and 12 h, no inhibitory rings (clear zones) were observed. A clear inhibitory zone appeared after 24 h, and its size did not change significantly after 48 h (Fig. 2). Therefore, the optimal incubation time was confirmed as 48 h.

### Culture Temperature

**Fig. 3:**
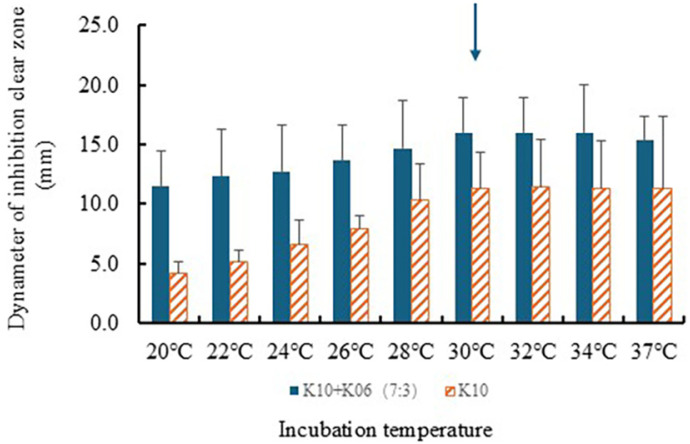
Effect of temperature on antibacterial activity in LAB co-cultures. Serial temperatures ranging from 20°C to 37°C were tested to determine their impact on the inhibition clear zone in mixed cultures of *Lb. plantarum* LBP-K10 and *Leu. mesenteroides* LBP-K06. The largest inhibition zone was observed at 30°C (indicated by arrow), establishing this as the optimal culture temperature.

Culture temperature is considered an important factor for the antibacterial effect of the LAB culture medium. System temperature profoundly affects the production of mixed culture systems and the activation energy of the bioreaction [28]. Serial temperatures from 20°C to 37°C were tested during LAB culture. A larger inhibition clear zone was confirmed in samples incubated at 30°C when repeated experiments were performed by temperatures in a mixed culture (Fig. 3). Therefore, the optimal culture temperature was confirmed as 30°C.

### Culture Methods: Stationary vs. Shaking

**Fig. 4:**
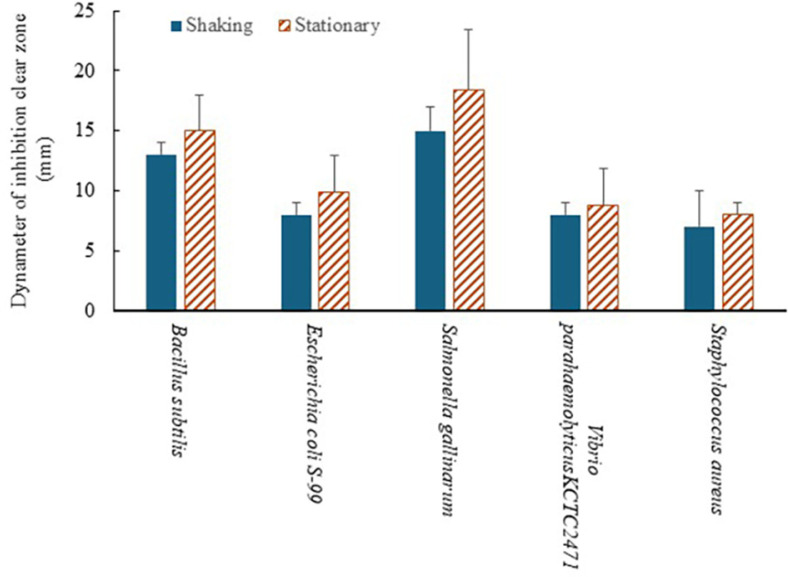
Comparison of antibacterial activity between stationary and shaking cultures. Samples from both stationary and shaking cultures (K10:K06 = 7:3), incubated at 30°C for 48 h, were analyzed using the disk diffusion assay. The stationary culture (indicated as solid black bars) produced a larger inhibitory zone compared to the shaking culture (indicated as hatched bars), indicating superior antibacterial activity in stationary conditions.

Samples from the stationary and shaking cultures (K10:K06 = 7:3), that were cultured in 30°C for 48 h, were subjected to a disc diffusion assay to compare the differences between the two culture methods in antibacterial activity. Larger clear inhibitory zones were identified in the stationary culture samples (Fig. 4), which exhibited better antibacterial activity than the shaking culture samples.

### Nutrient Supplementation

**Fig. 5:**
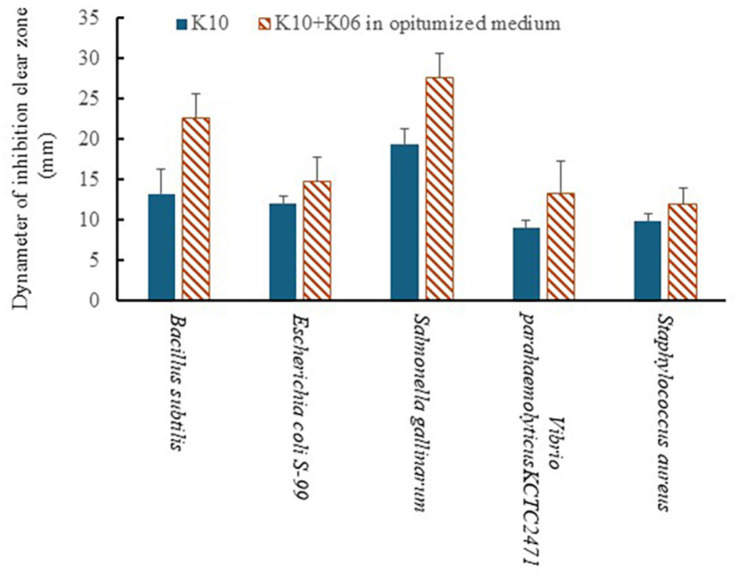
Enhanced antibacterial activity with amino acid and carbon sources supplementation. *Lb. plantarum* LBP-K10 and *Leu. mesenteroides* LBP-K06 were cultured at a 7:3 ratio in a stationary culture at 30°C for 48 h, supplemented with 4% sucrose and 1.5% mixed amino acids (indicated as hatched bars). This supplementation markedly increased antibacterial activity when compared with K10 cultured in conventional medium (indicated as solid black bars).

For better antibacterial activities of the LAB co-culture medium (K10:K06 = 7:3; stationary cultured for 48 h at 30°C), the nitrogen and carbon sources of the medium were further adjusted. In the case of carbon source supplementation, glucose, sucrose, and molasses showed larger clear inhibition zones with increasing concentrations, but the greatest inhibition ring was observed when the medium was prepared with 4% sucrose (Table 1). Therefore, the addition of 4% sucrose resulted in the highest antibacterial activity. Moreover, when mixed amino acids at 1.5% were added, the largest clear inhibitory zone was confirmed. In addition, although only a small amount of the mixed amino acids was added, a clear inhibitory zone was observed (Table 2). Therefore, adding 1.5% mixed amino acids resulted in the best antibacterial activity. Next, we cultured the K10 and K06 bacteria at a ratio of 7:3 in a stationary culture at 30°C for 48 h with 4% sucrose and 1.5% mixed amino acid supplementation, and the antibacterial activity was increased as shown in Fig. 5.

**Fig. 6:**
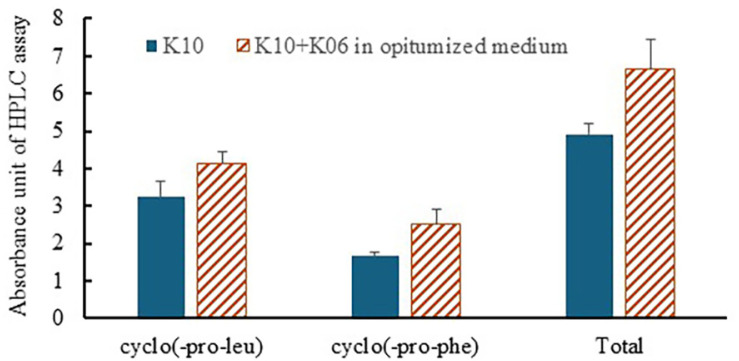
Production of cyclo(Leu-Pro) and cyclo(Phe-Pro) in LAB co-cultures. Following the identification of cyclo(Leu-Pro) and cyclo(Phe-Pro) as the primary active substances in LAB cultures, we confirmed their production within the cultures. *Lb. plantarum* LBP-K10 and *Leu. mesenteroides* LBP-K06 were co-cultured at a 7:3 ratio under stationary conditions at 30°C for 48 h, supplemented with 4% sucrose and 1.5% mixed amino acids. The total production of cyclo(Leu- Pro) and cyclo(Phe-Pro) in the co-culture (indicated as hatched bars) was 30% higher than that in the *Lb. plantarum* LBP-K10 mono-culture (indicated as solid black bars).

Having identified cyclo(Leu-Pro) and cyclo(Phe-Pro) as the primary active substances in LAB cultures, we further confirmed the production of these cyclodipeptides within the cultures. *Lb. plantarum* LBP-K10 and *Leu. mesenteroides* LBP-K06 were co-cultured (K10:K06 = 7:3) as a stationary culture at 30°C for 48 h with 4% sucrose and 1.5% mixed amino acid supplementation, and the total production of cyclo(Leu-Pro) and cyclo(Phe-Pro) was 30% higher than that of the *Lb. plantarum* LBP-K10-alone culture (Fig. 6).

In this study, the optimized culture conditions of the LAB co-culture system were established as showing that *Lb. plantarum* LBP-K10 and *Leu. mesenteroides* LBP-K06, co-cultured at a ratio of 7:3 using a stationary method for 48 h at 30°C, with 1.5% mixed amino acids and 4% sucrose added as nitrogen and carbon sources, exhibited the best antibacterial activity and the highest CDP production.

## Discussion

The research results presented in this article demonstrate the optimization of LAB (lactic acid bacteria) culture conditions to enhance antibacterial activity, with particular focus on cyclo(Leu-Pro) and cyclo(Phe-Pro) production. Our study elucidates that while mono-culture of *Lb. plantarum* LBP-K10 demonstrated baseline antibacterial activity, dual-strain co-cultivation significantly enhanced cyclic dipeptide (CDP) yields and antimicrobial efficacy. This synergy may arise from three interconnected mechanisms: First, metabolic cross-feeding enables precursor exchange between the strains, activating secondary metabolite pathways—a phenomenon widely validated in microbial consortia. Second, quorum sensing (QS) plays a pivotal role, as both strains efficiently secrete cyclo(Leu-Phe), a CDP molecule with demonstrated cell-proliferative and antimicrobial properties. This molecular crosstalk facilitates coordinated population-level behavior, amplifying CDP biosynthesis. Third, microenvironmental optimization occurs through dynamic regulation of pH and redox potential in the co-culture system. The shared acid tolerance and biofilm-forming capabilities of both strains in MRS medium provide a foundation for symbiotic adaptation, fostering stable biofilm architectures conducive to CDP synthesis. Although we did not conduct CFU or cell count measurements, our observations confirmed that both strains grew well together in the co-culture, with no significant dominance of one strain over the other throughout the culture period. This balanced interaction further supports the enhanced production of CDPs in the co-culture system.

Culture condition optimization revealed critical parameters governing CDP synthesis. A 48-h static incubation at 30°C emerged as the optimal protocol, balancing biomass proliferation (0– 24 h) with metabolic flux redirection toward CDP synthesis (24–48 h). Temperature modulation experiments confirmed 30°C as the ideal compromise for enzymatic stability and metabolic throughput, with deviations causing either protein denaturation or reduced catalytic efficiency. Static cultivation outperformed agitated methods by minimizing shear stress and promoting biofilm development, thereby creating a sheltered niche for metabolite accumulation. Nutrient supplementation with 4% sucrose and 1.5% mixed amino acids synergistically enhanced CDP yields and antibacterial activity, where sucrose fueled energy metabolism and amino acids directly supplied CDP precursors through optimized carbon-to-nitrogen stoichiometry.

The practical implications of this work span multiple industries. In food preservation, CDPs offer a natural, non-toxic alternative to synthetic preservatives, aligning with the global demand for clean-label products. For probiotic development, CDPs may enhance colonization competence and modulate gut microbiota through dual antibacterial and immunoregulatory actions. Notably, the unique mechanism of action of CDPs, which is distinct from that of conventional antibiotics, positions them as promising lead compounds for next-generation antimicrobials to combat drug-resistant pathogens. Specifically, their potent activity against *S*. Gallinarum ATCC 9184 highlights applications in poultry farming, where CDP-based feed additives could replace growth-promoting antibiotics to mitigate avian cholera outbreaks while curbing antimicrobial resistance transmission.

By integrating mechanistic insights with bioprocess optimization, this study establishes a scalable platform for CDP biosynthesis while bridging the gap between laboratory discovery and industrial implementation. These advances provide a robust foundation for developing novel biopreservatives, functional probiotics, and precision antimicrobial therapies, underscoring both scientific innovation and translational potential.

## Figures and Tables

**Table 1:** Antibacterial activities depending on the addition of various concentrations of nitrogen sources (Indicator strain: *Salmonella* Gallinarum ATCC 9184).

Nitrogen source	Clear zone size (mm)*					
	+0%	+0.5%	+1.0%	+1.5%	+2.0%	+2.5%
Yeast extract	0.0 ± 0.0	0.0 ± 0.0	9.0 ± 0.8	14.7 ± 2.0	15.1 ± 2.8	15.2 ± 4.1
Mixed amino acids	0.0 ± 0.0	12.8 ± 1.3	13.2 ± 1.2	16.5 ± 4.0	13.8 ± 3.8	14.1 ± 2.8
Soy peptone	0.0 ± 0.0	0.0 ± 0.0	12.3 ± 1.9	8.0 ± 0.0	16.3 ± 3.9	13.7 ± 1.2
Corn steep powder	0.0 ± 0.0	0.0 ± 0.0	0.0 ± 0.0	0.0 ± 0.0	0.0 ± 0.0	0.0 ± 0.0

*The values represent the average of three independent experiments.

**Table 2:** Antibacterial activities depend on the addition of various concentrations of carbon sources (Indicator strain: *Salmonella* Gallinarum ATCC 9184).

Carbon source	Clear zone size (mm)			
	+1%	+2%	+3%	+4%
Glucose	0.0 ± 0.0	9.5 ± 1.1	10.8 ± 1.3	11.8 ± 1.4
Sucrose	0.0 ± 0.0	10.8 ± 0.2	12.3 ± 1.2	13.5 ± 1.5
Fructose	0.0 ± 0.0	0.0 ± 0.0	0.0 ± 0.0	10.7 ± 0.9

*The values represent the average ± SD of three independent experiments.
